# Acute Pancreatitis as a Complication of an Intragastric Balloon

**DOI:** 10.7759/cureus.38094

**Published:** 2023-04-25

**Authors:** Laura Akiki, Abdallah Alomary

**Affiliations:** 1 Department of Gastroenterology, Al Zahraa Hospital University Medical Center, Beirut, LBN; 2 Department of Gastroenterology, Lebanese University Faculty of Medicine, Beirut, LBN

**Keywords:** pancreatitis, balloon, gastric, weight loss, endoscopy, gastrointestinal

## Abstract

The use of intragastric balloons (IGBs) for weight loss has increased in popularity over the past three decades. While they are generally considered effective and safe, there have been reports of various complications ranging from mild to severe. Acute pancreatitis is a rare complication following IGB insertion. In this case report, we describe the occurrence of acute pancreatitis in a patient six months after IGB insertion (ORBERA, Apollo Endosurgery, Texas, USA). The balloon was found to be in the appropriate position and was removed endoscopically, leading to rapid clinical and biological improvement.

## Introduction

Obesity is a pandemic metabolic illness that can lead to various serious diseases [1]. Intragastric balloon (IGB) therapy is gaining popularity as a minimally invasive and temporary technique for promoting weight loss in obese patients with a body mass index (BMI) ranging between 30 and 40 kg/m^2^ [2]. It is considered safe and efficient [3]. The most common adverse events associated with IGB are mild, including nausea, vomiting, and decrease oral intake, and are usually managed conservatively. Other complications leading to balloon removals such as abdominal pain or obstruction of the digestive tract occur less frequently. More serious adverse events such as death or perforation are rarely reported [3,4]. Here, we describe the case of a patient who presented with acute pancreatitis as a rare complication of IGB placement.

## Case presentation

**Figure 1:**
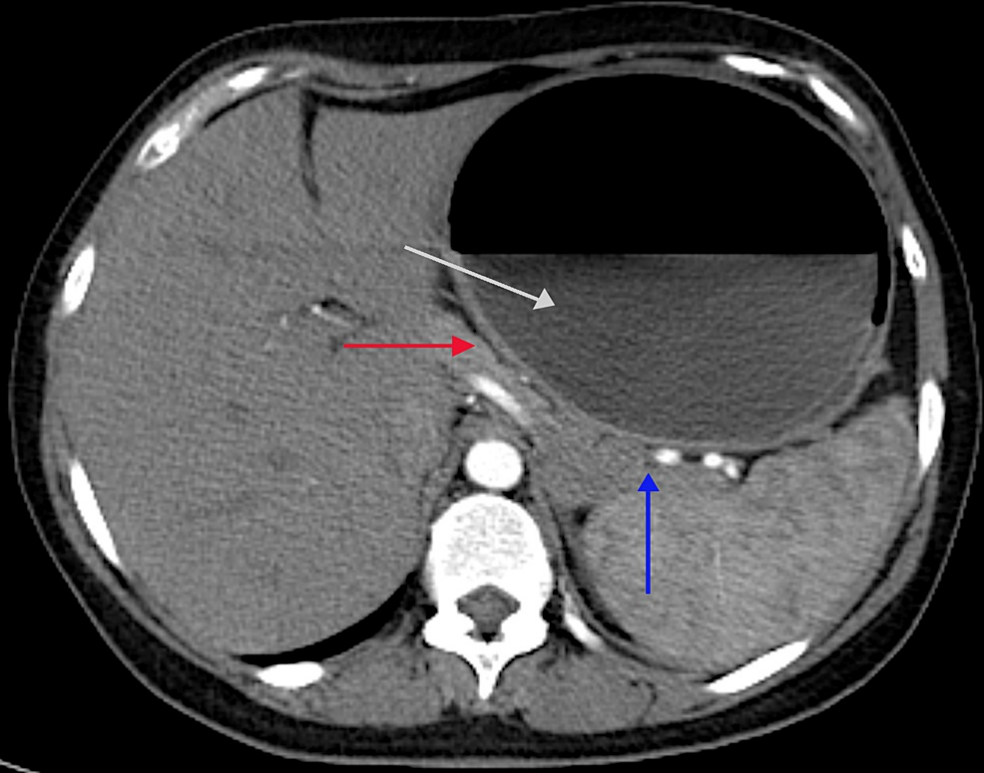
Computed tomography scan of the abdomen in the axial plane, showing the over-distended balloon half filled with fluid and containing air-fluid level (white arrow) compressing the pancreatic head (red arrow) with fluid and fat stranding surrounding the pancreatic tail (blue arrow).

**Figure 2:**
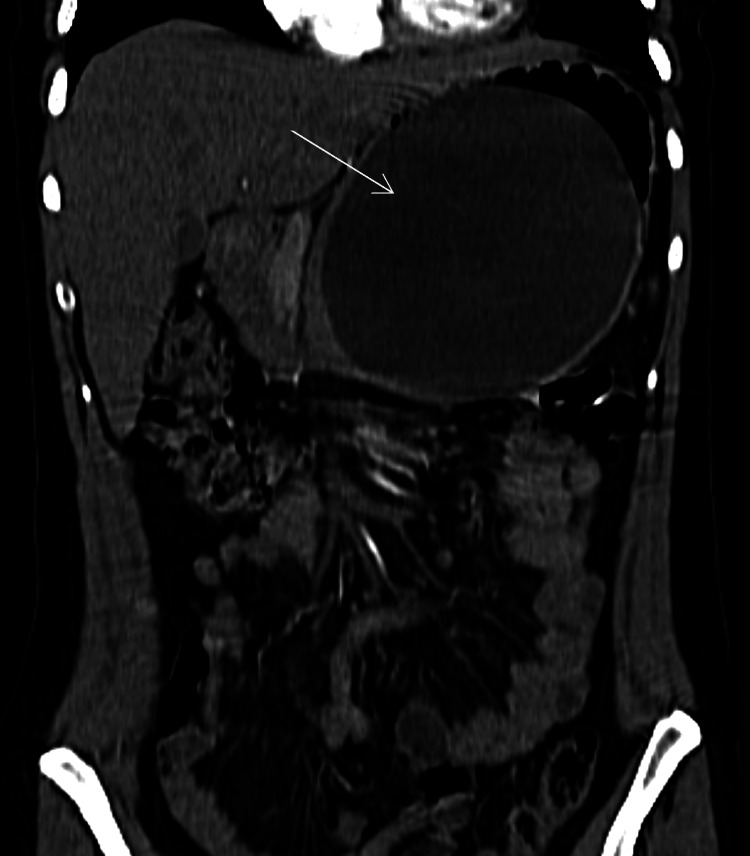
Computed tomography scan of the abdomen in the coronal plane, showing the over-distended intragastric balloon in its correct gastric location (white arrow).

A 37-year-old woman presented to the emergency department with severe epigastric pain of one-week duration. The pain was continuous, moderately severe, started suddenly, and persisted despite symptomatic treatment. It radiated to the back and was accompanied by nausea, vomiting, and a decrease in oral intake. There were no other associated symptoms. The patient was a non-smoker with no history of alcohol consumption. Her medical history included hypertension, which was well-controlled with bisoprolol, and obesity (BMI = 35 kg/m^2^), for which she underwent IGB insertion (ORBERA, Apollo Endosurgery, USA, Texas) six months ago for weight loss (current BMI = 30 kg/m^2^). She denied any recent abdominal trauma or new drug use. Her family history was insignificant. The clinical evaluation revealed a mildly tachycardic patient, who was otherwise stable, and an isolated severe epigastric tenderness on abdominal examination. Lab tests showed mild leukocytosis without C-reactive protein elevation, normal renal and liver function tests, and an elevated lipase level four times the upper limit of normal (435 IU/L). The extended analysis showed serum calcium and triglyceride levels within the normal range. We performed an abdominal CT scan which showed an IGB that was over-distended with an approximate volume of 1,140 mL (14 × 11 × 14 cm), half-filled with fluid and displaying an air-fluid level. At the level of the pancreas, the body and tail were compressed by the IGB, with some fluid below the tail, suggesting mild acute pancreatitis (Figure 1). The CT severity index was 3. The coronal view showed that, despite being over-distended, the IGB was in its correct gastric location (Figure 2). The liver, gallbladder, and intrahepatic bile ducts were intact.

The patient was diagnosed with acute pancreatitis (bedside index of severity in acute pancreatitis (BISAP) score = 0) secondary to extrinsic pancreatic compression by IGB. She was hospitalized and started on intravenous hydration, painkillers, and an antiemetic. Urgent upper gastrointestinal endoscopy was performed. The balloon was in the correct gastric position and was deflated and removed without complications. The gastric mucosa was inspected and intact. The patient began to improve immediately following the balloon removal. Her pain was relieved and she started to tolerate oral intake. She was discharged home on day two free of symptoms. The scheduled follow-up one week later was unremarkable.

## Discussion

The technology of IGB has not stopped evolving since its invention four decades earlier. Currently, there are various types available in the market that differ in their characteristics [5] but share good tolerability and efficacy [3,4]. They are considered safe as long as the manufacturer’s recommendations are followed. The most commonly associated adverse events are mild and limited to nausea/vomiting (23.3%) and abdominal pain (19.9%). However, some serious complications have been reported on rare occasions such as death (0.05%) and gastric perforation (0.1%) [6].

Acute pancreatitis following IGB is rare. Only a few case reports were described in the literature before the Food and Drug Administration (FDA) issued a warning in 2016. Since the FDA warning was issued in 2016, the agency has received reports of over 30 cases of acute pancreatitis [7]. The exact incidence is unknown, and the underlying mechanism is believed to be secondary to the mass effect of the balloon on the pancreatic parenchyma and/or duct, or the dislodgement of the catheter into the second part of the duodenum [8]. Halpern et al. [9] reported a unique case of asymptomatic lipase elevation secondary to IGB insertion that was observed on day two and persisted until balloon removal in the third month. The authors suggested that the asymptomatic elevation in pancreatic enzymes could be a precocious marker of IGB-induced acute pancreatitis. The timing of occurrence of acute pancreatitis is variable and ranges from one day to 11 months after the IGB insertion [6]. While IGB-induced acute pancreatitis has been frequently reported to be mild, some patients may experience more serious manifestations such as peripancreatic fluid collections or pancreatic necrosis. This further emphasizes the importance of early diagnosis to prevent potentially severe sequelae [8].

Similarly, spontaneous hyperinflation has been also reported by the FDA as a potential complication of fluid-filled IGB. Patients should be informed and educated about this potential complication [10]. Its exact incidence is unknown and its timing is unpredictable. The underlying mechanism is yet to be identified, although balloon permeability or gas-producing infectious microorganisms [11] have been evoked in some cases. Hyperinflated balloons can be discovered incidentally at the time of the scheduled balloon removal if asymptomatic [10] or can present with gastric outlet obstruction [12]. If left in place, gastric volvulus [13] or necrosis [14] may result.

Acute pancreatitis in the context of spontaneous hyperinflation of the fluid-filled balloons is a rarer presentation [15]. Pancreatitis is believed to be secondary to the compressive effect of the over-distended stomach. In addition to the standard of care, the management of complications related to IGB typically involves balloon removal. IGB-induced acute pancreatitis is not an exception to this rule. The endoscopic approach should be attempted first. Surgical removal was considered in rare cases where endoscopic retrieval was infeasible [16,17]. On the other hand, in some cases of IGB-induced pancreatitis, clinical and laboratory improvements were achieved with conservative management alone [9,17]. Partial deflation of the symptomatic hyperinflated balloon has been proposed for pressure release if the device allows it [15].

## Conclusions

Our patient developed a mild case of acute pancreatitis secondary to the IGB placement. This complication occurred at the end of the intended placement duration of six months, despite following manufacturer instructions. The balloon was in its correct gastric location but was found to be hyperinflated and containing an air-fluid level. The extrinsic compression of the pancreas by the distended stomach is believed to be the etiology of acute pancreatitis. The endoscopic removal of the balloon led to rapid clinical improvement and correction of the biochemical abnormalities. Despite their low incidence, clinicians should be aware of acute pancreatitis and hyperinflation as potential complications of IGB placement. Prompt management should be done to avoid severe consequences. The role of asymptomatic elevation in pancreatic enzymes as an early marker for the prediction of this complication should be explored in subsequent research.
